# Exploring the Therapeutic Potential of DPP4 Inhibitors in Alzheimer's Disease: Molecular Insight and Clinical Outcome

**DOI:** 10.7759/cureus.72648

**Published:** 2024-10-29

**Authors:** Albert Joseph Sulangi, Sarah E Lyons, Amy A Abdou, Hemangi Patel, Deepika Nagliya, Eileen Joseph, Carmel Joseph, Divya Kumar, Shivani Patel, Isha Jinwala, Mayur S Parmar

**Affiliations:** 1 Osteopathic Medicine, Nova Southeastern University Dr. Kiran C. Patel College of Osteopathic Medicine, Clearwater, USA; 2 Osteopathic Medicine, Nova Southeastern University Dr. Kiran C. Patel College of Osteopathic Medicine, Fort Lauderdale, USA; 3 Osteopathic Medicine, Nova Southeastern University, Fort Lauderdale, USA; 4 Foundational Sciences, Nova Southeastern University Dr. Kiran C. Patel College of Osteopathic Medicine, Clearwater, USA

**Keywords:** alzheimer’s dementia, amyloid plaques, dipeptidyl peptidase-4 inhibitors, drugs repurposing, neurofibrillary tangles (nfts), types 2 diabetes

## Abstract

Alzheimer’s disease (AD) is a progressive neurodegenerative disorder characterized by amyloid-beta (Aβ) plaques, neurofibrillary tangles, and cognitive decline. Given the shared neuropathophysiological traits between AD and type 2 diabetes mellitus (T2DM), repurposing antidiabetic medications, such as dipeptidyl peptidase 4 inhibitors (DPP4i), has emerged as a promising therapeutic strategy. This review comprehensively evaluates the preclinical and clinical evidence supporting the potential of DPP4i in preventing or treating AD by modulating Aβ and tau pathology, improving cognitive function, reducing neuroinflammation and oxidative stress, and promoting neuronal survival. The beneficial effects of DPP4i are likely mediated through the modulation of insulin signaling, anti-inflammatory and antioxidant properties, glucagon-like peptide-1 (GLP-1) upregulation, and modulation of the amyloidogenic pathway. While further research is needed to establish their clinical efficacy in AD patients, DPP4i offers a promising avenue for therapeutic intervention for this devastating neurodegenerative disease.

## Introduction and background

Dementia is a general term for a decline in cognitive function severe enough to interfere with daily life. It encompasses a range of symptoms affecting memory, thinking, and social abilities. Although dementia primarily affects older adults, it is not a normal part of aging. Alzheimer’s disease (AD) is the most prevalent form of dementia (60-80% of all cases of dementia). It is a progressive neurological disorder resulting in significant memory impairment and cognitive decline in patients and impacts over 6.9 million Americans aged 65 and above [1]. The underlying hallmark pathology involves the accumulation of amyloid β (Aβ) plaques and neurofibrillary tangles (NFTs), leading to impaired neuronal function [1,2].

Diabetes mellitus (DM), particularly type 2 diabetes mellitus (T2DM), is a chronic metabolic disorder characterized by insulin resistance and hyperglycemia, which can lead to complications like nephropathy, retinopathy, neuropathy, and cardiovascular diseases [3-5]. T2DM patients have an increased risk of cognitive impairment and AD [1,5]. Notably, both AD and T2DM have the highest prevalence in the elderly population, with individuals suffering from T2DM facing an increased risk of cognitive decline and dementia, including mild cognitive impairment (MCI) and AD [6]. Comorbidities often associated with T2DM, such as cardiovascular disease and hypercholesterolemia, are also major common risk factors for developing AD [7-9]. In both these conditions, shared risk factors and mechanisms, such as disrupted insulin signaling, glucose metabolism, increased dipeptidyl peptidase 4 (DPP4) activity, and reduced GLP-1 activity, contribute to the aggregation of tau and amyloid proteins, driving neurodegeneration [1,5].

Dipeptidyl peptidase 4 inhibitors (DPP4i), such as sitagliptin, saxagliptin, linagliptin, and alogliptin, are a class of oral hypoglycemic agents used in the management of T2DM. Although not considered first-line therapy, they can be used as an add-on treatment when lifestyle modifications and metformin fail to control blood glucose levels [10]. DPP4i can also be used as monotherapy in patients who are intolerant to or have contraindications to metformin, such as those with chronic kidney disease [11]. These medications inhibit the DPP4 enzyme, which degrades incretin hormones like GLP-1, leading to enhanced insulin release, increased GLP-1 activity, and reduced glucagon secretion, thereby improving glycemic control [12]. Inhibition of DPP4 aids in prolonging the half-life of GLP1, which helps potentiate insulin release. Beyond glucose homeostasis, selective GLP-1 agonists have shown neuroprotective effects by mitigating AD neuropathology in preclinical studies and improving cognitive function in both preclinical and clinical studies, suggesting a potential influence on AD pathophysiology [1,13].

Currently, the available medications offer limited symptomatic control and have no disease-preventative role in AD [14]. FDA-approved medications like memantine and cholinesterase inhibitors provide marginal and non-sustained benefits [1,2]. Recently approved monoclonal antibodies, such as lecanemab and donanemab, reduce amyloid pathology and show some clinical benefits in reducing cognitive impairment, but these medications carry an increased risk of amyloid-related imaging abnormality (ARIA). This risk is higher in homozygous carriers of apolipoprotein e4 (APOE e4), including those with comorbid vascular pathology [2]. Clinically, there is interest in repurposing existing medications, including antidiabetic medications, as disease-modifying therapies targeting underlying pathological mechanisms of AD, such as amyloid plaque burden, neurofibrillary tangles, neuroinflammation, and mitochondrial dysfunction [1]. These repurposed medications could have a lower risk of severe adverse effects and, when used early, may help prevent or delay AD progression. Given the significant overlap between T2DM and AD and the emerging evidence suggesting a potential neuroprotective role for DPP4i, exploring their therapeutic potential in AD could provide new avenues for disease management.

This comprehensive review aims to evaluate existing preclinical and clinical research on the pharmacologic efficacy and effectiveness of DPP4i in preventing AD neuropathology and disease progression. In addition, this review evaluates the potential molecular mechanisms underlying DPP4i’s therapeutic potential. It adds to the existing literature by providing a detailed analysis of the current evidence, identifying inconsistencies, and proposing new avenues for research, such as the evaluation of the long-term effects of DPP4i on cognitive function in AD patients and the potential for DPP4i to be integrated into existing treatment regimens for AD.

## Review

AD is a debilitating neurodegenerative disorder characterized by the presence of amyloid-beta (Aβ) senile plaques, NFTs (hyperphosphorylated tau protein), and a decline in neuronal and synaptic function in the brain. There are shared neuropathophysiological traits between AD and T2DM, such as insulin resistance, dysfunctional insulin signaling, synaptic dysfunction, neuroinflammation, mitochondrial and autophagic impairments, and altered glucose metabolism [15]. This suggests that medications used to treat T2DM might be beneficial in treating AD. One class of antidiabetic medications, DPP4i, increases GLP-1 levels and has shown neuroprotective effects. Table 1 below summarizes some of the findings from the preclinical and clinical observational studies highlighting the impact of DPP4i on AD-associated neuropathophysiological and cognitive impairment. 

**Table 1:** Summarizes the preclinical and clinical observational studies highlighting the effects of DPP4i on AD-associated neuropathophysiological and cognitive impairment. T2D-AD: Type 2 Diabetes-Alzheimer's Disease; NORT: Novel Object Recognition Test; MWM: Morris Water Maze; IRS1: Insulin Receptor Substrate 1; Akt: Protein kinase B; GSK3β: Glycogen synthase kinase 3 beta; ICV: Intracerebroventricular; GLP-1: Glucagon-like peptide-1; GLP-1R: Glucagon-like peptide-1 receptor; TNF-α: tumor necrosis factor alpha; IL-6: Interleukin 6; IL-1β: Interleukin 1 beta; TBARS: thiobarbituric acid reactive substances; CAT: Catalase; AchE: Acetylcholinesterase; H&E: Hematoxylin and eosin, BACE-1: Beta-secretase 1; NFs: Neurofilaments; PI3K: Phosphoinositide 3-kinase; SAP97: Synapse-associated protein 97; PSD95: Postsynaptic density protein 95; SYN-1: Synapsin I; CREB: cAMP response element-binding protein; eNOS: endothelial nitric oxide synthase; GIP: glucose-dependent insulinotropic polypeptide; GFAP: glial fibrillary acidic protein; RAM: Radial Arm Maze; βAPP: beta-amyloid precursor protein; DPP4i: dipeptidyl peptidase-4 inhibitor; MMSE: Mini-Mental State Examination; APOE: apolipoprotein E; AD: Alzheimer's Disease

Study	Drug	Experimental model	Key Findings
Sim et al., 2023 [16]	Sitagliptin	T2D-AD mouse model (High-fat diet feed mice administered with a single streptozotocin dose intraperitoneally)	Sitagliptin: ✔️Improved cognitive function: Significantly improved hippocampal-dependent learning, memory, and cognitive functions in the T2D-AD mouse model, as demonstrated by behavioral tests, the Novel object recognition test (NORT), and the Morris water maze (MWM). ✔️Improved brain insulin signaling via the IRS1/Akt/GSK3β pathway, thereby, reduced: Aβ accumulation by increasing insulin-degrading enzyme levels and hyperphosphorylated Tau (pTau) levels. ✔️Improved insulin sensitivity. ✔️Reduced body weight. ✔️Ameliorated inflammation and promoted the metabolic gene expression in the liver.
Siddiqui et al., 2021 [17]	Linagliptin	AD rats model induced with Aβ (1−42) peptides	Linagliptin: ✔️Cognitive and motor improvement: Significantly reversed motor and cognitive impairments in the rats, as assessed through locomotor activity (LA) and MWM tests. ✔️Attenuated the soluble Aβ (1−42) senile plaque deposition in the hippocampus. ✔️Ameliorates ICV-Aβ (1−42) peptides-induced motor impairment in locomotor activity. ✔️Increased GLP-1 Levels in the hippocampus. ✔️Decreased insulin resistance: Reduced insulin resistance in the hippocampus by decreasing serine phosphorylation (ser307) of insulin receptor substrate-1 (IRS-1). ✔️Reduced inflammatory markers: Lowered TNF-α, TNF-α, IL-6 and IL-1β levels. ✔️Reduced oxidative stress markers, such as TBARS, nitrite, and CAT in the hippocampus.✔️Prevented hippocampal GSK-3β stimulation dose-dependently. ✔️Attenuated acetylcholinesterase (AchE) activity in the hippocampus. ✔️Neuroprotective and anti-amyloidogenic effects as evidenced by histopathological analysis using H&E and Congo red staining.
Rahman et al., 2020 [18]	Alogliptin	AD rats model induced with Aβ (1−42) fibrils	Alogliptin: ✔️Attenuated Aβ (1-42)-induced cognitive impairment: Significantly restored cognitive functions in AD-induced rats, as evidenced by their Morris Water Maze (MWM) test performance. ✔️Attenuated Aβ (1-42)-induced insulin resistance: Significantly reduced insulin levels and IRS-1pS307 expression, improving hippocampal insulin resistance. ✔️Decreased GSK-3β activity in the hippocampus. ✔️Reduced inflammatory and oxidative stress markers: Lowered the levels of TNF-α and oxidative stress (malondialdehyde level) in the hippocampus. ✔️Reduced Aβ (1–42) fibrils-induced hippocampal glutathione (GSH) levels. ✔️Enhanced GSH levels. ✔️Neuroprotective and anti-amyloidogenic effects: Histopathological analysis supported alogliptin's ability to attenuate neuronal damage and amyloidosis. ✔️Binding affinity: In-silico analysis showed that alogliptin has a good binding affinity with Aβ and beta-secretase-1 (BACE-1), suggesting a potential mechanism for its effects.
Chen et al., 2019 [19]	Sitagliptin and Saxagliptin	3xTg AD mice	Sitagliptin and Saxagliptin: ✔️ Improved GLP-1 and GLP-1R levels: Both sitagliptin and saxagliptin improved GLP-1 and GLP-1 receptor expression levels in the hippocampus and cortex of transgenic AD mice. ✔️Enhanced cognitive function: Protected learning and memory, as evidenced by improved MWM test performance. ✔️Improved spatial learning and memory. ✔️Reduced Tau and neurofilament phosphorylation: Sitagliptin and saxagliptin improved abnormal phosphorylation and O-glycosylation of tau and neurofilament (NFs) proteins, promoting Tau binding to microtubules. ✔️Reduced PI3K/AKT/GSK3 pathway. ✔️Decreased Aβ accumulation: Increased Aβ degradation, reducing its accumulation in the brain. ✔️Alleviated neurodegeneration. ✔️Upregulated synapse protein levels: Upregulated the expression of synapse proteins (SAP97, PSD95, SYN-1, and SP1) and activation of CREB, which are crucial for synaptic function and memory.
Nakaoku et al., 2019 [20]	Linagliptin	PS1 tauopathy AD model	Linagliptin: ✔️Restored spatial reference memory as assessed by MWM. ✔️No effect on Tau phosphorylation in the hippocampus. ✔️Increased GLP-1 levels. ✔️Ameliorated high-fat diet-induced hyperglycemia. ✔️Decreased fasting blood glucose. ✔️Increased cerebral blood flow. ✔️No Effect on eNOS.
Kosaraju et al., 2017 [21]	Linagliptin	3xTg-AD Mouse Model	Linagliptin: ✔️Improved cognitive performance: Mitigated cognitive deficits in the mice, as evidenced by performance in the MWM and Y-maze tests. ✔️Reduced Aβ42 but not Aβ40. ✔️Reduced amyloid plaque deposition. ✔️Decreased Tau phosphorylation (Ser202/Thr205). ✔️Increased brain GLP-1 and GIP levels. ✔️No effect on plasma glucose levels. ✔️Reduced neuroinflammation as evidenced by a reduction in GFAP.
Kosaraju et al., 2013 [22]	Vildagliptin	Streptozotocin-induced rat AD model	Vildagliptin: ✔️Improved memory retention: Reversed the reference and working memory deficits in the radial arm maze task and the spatial learning deficits in the hole-board task. ✔️Reduced Aβ42 levels in the hippocampus and cortex. ✔️Decreased Tau phosphorylation. ✔️Neuroprotection: Prevented neuronal loss in the hippocampus at the CA1 region and the cortex at layers II-IV. ✔️Increased GLP-1 levels in the hippocampus and cortex. ✔️Reduced inflammatory markers: TNF-α and IL-1β levels.
Kosaraju et al., 2013 [23]	Saxagliptin	Streptozotocin-induced rat AD model	Saxagliptin: ✔️Enhanced the reference and working memory deficits in the RAM task. ✔️Reduced Aβ42 levels. Decreased total and phosphorylated Tau. ✔️Neuroprotection: Mild neurodegeneration was observed with good and dense neuronal density (H and E staining) and increased CV-positive neurons compared to the negative control group. ✔️Reduced inflammatory markers (TNF-α and IL-1β levels). ✔️Improved GLP-1 levels in the hippocampus.
D'Amico et al., 2010 [24]	Sitagliptin	B6.Cg-Tg(APPswe,PSEN1dE9)85Dbo/J	Sitagliptin: ✔️Improved memory function: Counteracted memory impairment in the contextual fear conditioning test, indicating improved memory-related behavioral paradigms. ✔️Decreased the number and total area of βAPP and Aβ deposits Increased GLP-1 levels. ✔️Reduced nitrosative stress and neuroinflammation within the brain. ✔️Dose-dependent effects were much more evident at the highest dose of 20 mg/kg sitagliptin.
Zullo et al., 2022 [25]	DPP4i and sulfonylureas	Nursing home residents	DPP4i class: ✔️The study evaluated the effects of DPP4i and sulfonylureas on cognitive and physical function in nursing home residents.
Secnik et al., 2021 [26]	Multiple antidiabetic medications	Patients with diabetes and dementia (Prospective Open-Cohort Study)	DPP4i class: ✔️DPP4i attenuated the decline in MMSE scores. Compared to DPP4i, insulin and sulfonylureas had larger point decrements in MMSE scores.
Wu et al., 2020 [27]	Metformin or DPP4i	People with T2DM with normal cognition or AD	DPP4i class: ✔️DPP4i slow memory decline in Alzheimer's, especially in APOE ε4 carriers, with benefits linked to reduced inflammation and amyloid-beta levels.
Chen et al., 2020 [28]	DPP4i	Patients with type 2 diabetes	DPP4i class: ✔️DPP4i decreased the risk of dementia with a class effect, especially vascular dementia, but not in AD.
Kim et al., 2018 [29]	DPP4i and sulfonylureas	Older patients with type 2 diabetes	DPP4i class: ✔️The study suggests that patients taking DPP4i had a lower risk of developing dementia compared to those taking sulfonylureas.
Isik et al., 2017 [30]	Sitagliptin	Elderly diabetic patients with or without AD (Prospective, open-label, randomized, controlled trial)	Sitagliptin: ✔️Showed potential benefits in improving cognitive functions in elderly diabetic patients. ✔️Cognitive improvements were observed in diabetic patients with and without AD.

Effects of DPP4 inhibitors on Aβ and tau pathology

DPP4i have shown promising therapeutic potential to modulate two key pathological hallmarks: Aβ burden and tau phosphorylation. Preclinical studies consistently demonstrate the efficacy of these inhibitors in mitigating these pathological processes, offering hope in evaluating these therapeutics early in the disease course in AD patients.

Aβ Reduction

DPP4i, such as saxagliptin, linagliptin, and alogliptin, have been shown to directly decrease Aβ42 levels, potentially through enhanced degradation or production inhibition [16-19,21,24,31,32]. Additionally, linagliptin has reduced amyloid plaque deposition [17,21,32]. Sitagliptin has exhibited effects on early Aβ production by decreasing β-amyloid precursor protein (βAPP) and Aβ deposits [24]. Linagliptin has also been shown to reduce BACE-1 and γ-secretase levels, further modulating the amyloidogenic pathway [18]. Vildagliptin has demonstrated anti-amyloidogenic potential by decreasing the size of HSA fibrillation and potentially decelerating amyloid production [33].

Tau Phosphorylation Inhibition

DPP4i have demonstrated the ability to reduce tau phosphorylation, thus mitigating the formation of neurofibrillary tangles. Linagliptin, vildagliptin, sitagliptin, and saxagliptin have been shown to reduce abnormal tau phosphorylation, potentially promoting its binding to microtubules and restoring normal function [16,19,21-23]. Additionally, they further support their role in combating tau-related neurodegeneration. However, in the tauopathy model, PS1, linagliptin did not affect the phosphorylation levels of tau, which requires further validation in other animal models [20]. A novel DPP4i, Gramcyclin A, has shown promise in decreasing hyperphosphorylated tau levels and Aβ plaque burden in AD mice [34].

The multifaceted effects of DPP4i on Aβ and tau pathology underscore their potential as disease-modifying agents for AD. By reducing Aβ burden and inhibiting tau phosphorylation, these inhibitors target two core pathological processes underlying AD. Additionally, the ability of DPP4i to increase GLP-1 levels, which in turn has been shown to decrease Aβ levels and exert neuroprotective effects through the modulation of insulin signaling and GSK-3β activity, further strengthens the potential of DPP4i in AD treatment [16,18,19,21-24]. While further research is necessary, preclinical evidence suggests DPP4i represents a valuable avenue for therapeutic intervention in the fight against this devastating neurodegenerative disease.

Effects of DPP4 inhibitors on cognitive function

As previously discussed, mitigating Aβ and tau pathology likely contributes to the observed improvements in cognitive function in preclinical studies. DPP4i have demonstrated promising results in reversing impaired spatial and learning memory, as assessed by various behavioral tests.

Preclinical Studies

For instance, sitagliptin significantly enhanced hippocampal-dependent learning, memory, and cognitive functions in a T2D-AD mouse model, as evidenced by the Novel Object Recognition Test (NORT) and Morris Water Maze (MWM) [16]. Similarly, linagliptin markedly reversed motor and cognitive impairments in AD rat models, as assessed through locomotor activity and MWM tests [17,20,32]. Alogliptin also mitigated Aβ-induced cognitive impairment in AD rats, as shown by improved MWM performance [18]. Furthermore, sitagliptin and saxagliptin enhanced cognitive function and protected learning and memory in 3xTg AD mice [19].

These cognitive improvements are likely linked to the reduction in Aβ and tau pathology and the modulation of other key pathways. For example, linagliptin's ability to increase GLP-1 levels, which promotes neurogenesis and reduces neuroinflammation, may contribute to its cognitive-enhancing effects [17,21,32,35]. Additionally, reducing insulin resistance and oxidative stress observed with several DPP4i could enhance cognitive function [18,32,36].

Clinical Studies

Clinical studies also support the potential cognitive benefits of DPP4i. Observational and randomized controlled trials have indicated that DPP4i can improve glucose control, protect against worsening cognitive function in older patients with T2DM and MCI, and reduce the risk of all forms of dementia, including AD [29,37,38]. For instance, vildagliptin has been associated with stabilized cognitive performance and protecting against worsening cognitive functioning in elderly diabetic patients with MCI, as measured by MMSE [39,40]. Preliminary data also hints that sitagliptin may improve cognitive function compared to metformin [40]. Furthermore, combining DPP4i with metformin might provide greater protection against AD than combining metformin with sulfonylureas. Meta-analyses have also indicated in T2DM patients a potential association between DPP4i use and a decreased risk of all-cause dementia and vascular dementia [37]. Interestingly, studies have also shown a correlation between increased DPP4 activity and impaired cognitive function in humans, suggesting a potential role for DPP4 inhibition as a promising approach to preserving cognitive abilities.

These preclinical and clinical findings collectively suggest that DPP4i offers a promising therapeutic avenue for targeting the underlying pathology of AD and improving cognitive function in patients with and without T2DM. Further research is needed to fully understand the mechanisms underlying these effects and evaluate the clinical efficacy of DPP4i in improving cognitive outcomes in AD patients, particularly in well-controlled clinical trials.

Effects of DPP4 inhibitors on neuroinflammation and oxidative stress

Beyond targeting the key hallmarks of AD and improving cognitive function, DPP-4 inhibitors also demonstrate a beneficial impact on neuroinflammation and oxidative stress [17,18,21,24,32]. In the pathogenesis of AD, chronic inflammation and oxidative stress play major contributing roles, sharing a common molecular pathophysiology observed in T2DM [41,42]. These findings could suggest that DPP4i's ability to mitigate inflammation and oxidative stress can contribute to reducing amyloid and tau pathology, or this observation could result from the reduction in amyloid and tau pathology.

For example, sitagliptin has been shown to alleviate inflammation and promote metabolic gene expression in the liver, suggesting a systemic anti-inflammatory effect that could extend to the brain [16]. Linagliptin also reduced inflammatory markers such as TNF-α, IL-1β, and IL-6, as well as oxidative/nitrosative stress markers in the hippocampus of AD rat models [17]. Similarly, alogliptin lowered the levels of TNF-α and oxidative stress in the hippocampus [18]. Linagliptin's ability to reduce neuroinflammation was further supported by a reduction in GFAP in a 3xTg-AD mouse model [21]. Both vildagliptin and saxagliptin also reduced inflammatory markers (TNF-α and IL-1β levels) in streptozotocin-induced rat AD models [22]. In addition, sitagliptin has also been shown to reduce nitrosative stress and inflammation in a B6.Cg-Tg(APPswe,PSEN1dE9)85Dbo/J mouse model [24].

These findings suggest that DPP-4 inhibitors may benefit cognitive function by directly targeting Aβ and tau pathology and modulating the neuroinflammatory response, which plays a crucial role in AD progression. Further research is needed to fully elucidate the complex interplay between DPP-4 inhibition, Aβ and tau pathology, and neuroinflammation in AD.

Neuroprotective effects of DPP4 inhibitors on neuronal survival

Beyond cognitive benefits, DPP4i also exhibits neuroprotective effects, as evidenced by neuronal survival in various brain regions. Preclinical studies have shown that these inhibitors can attenuate neuronal damage and promote neuronal survival, potentially contributing to their therapeutic potential in AD [18,19,22,23].

For example, vildagliptin has been shown to prevent neuronal loss in the hippocampus and cortex in a streptozotocin-induced rat AD model [22]. Similarly, saxagliptin demonstrated neuroprotective effects in a comparable model, with histopathological analysis revealing mild neurodegeneration and an increased number of viable neurons compared to the control group [23].

The mechanisms underlying the neuroprotective effects of DPP4i are likely multifaceted and may involve a combination of direct and indirect actions. As discussed earlier, reducing Aβ and tau pathology likely plays a crucial role in protecting neurons from damage. Additionally, the ability of DPP4i to modulate insulin signaling, reduce synaptic loss, reduce inflammation, and decrease oxidative stress may further contribute to their neuroprotective effects [16,18,19,21-24,31,32,36]. For instance, linagliptin's ability to increase GLP-1 levels has been linked to neuroprotection through the modulation of insulin signaling and the inhibition of GSK-3β, a key enzyme involved in tau hyperphosphorylation and neurotoxicity [17,32].

These findings suggest that DPP4i offers a promising therapeutic strategy for targeting the underlying pathology of AD, promoting neuronal survival, and protecting against neurodegeneration. Further research is needed to fully elucidate the mechanisms underlying these neuroprotective effects and evaluate their clinical relevance in AD patients.

Potential beneficial molecular mechanisms of DPP4 inhibitors in mitigating AD pathophysiology

The therapeutic benefits of DPP4i in AD appear to be associated with molecular changes, particularly in pathways linked to insulin signaling, inflammation, and oxidative stress.

Modulation of Insulin Signaling

DPP4i have been shown to enhance brain insulin signaling, primarily through the IRS1/Akt/GSK3β pathway, which is crucial for neuronal survival and function [16]. DPP4i may promote neurogenesis and protect against neurodegeneration by increasing insulin sensitivity and reducing insulin resistance [17,18,32].

Anti-inflammatory and Antioxidant Effects

As mentioned, DPP4i have demonstrated anti-inflammatory and antioxidant properties by reducing inflammatory markers (such as TNF-α, IL-1β, and IL-6) and oxidative stress markers. These effects may contribute to neuroprotection and improved cognitive function.

GLP-1 Upregulation

Several DPP4i, including linagliptin, vildagliptin, sitagliptin, and saxagliptin, have been shown to increase levels of GLP-1, a peptide hormone with known neuroprotective effects [16, 17, 19-24, 31]. An increase in the GLP-1 receptor expression has also been noted with sitagliptin and saxagliptin treatment [19]. GLP-1 has been shown to improve learning and memory, potentiate the insulin pathway, and reduce Aβ levels [1]. By increasing GLP-1 levels, DPP4i may indirectly contribute to enhanced cognitive function and reduced Aβ pathology.

Modulation of the Amyloidogenic Pathway

Some DPP4i, such as linagliptin, have been shown to reduce levels of BACE-1 and γ-secretase, key enzymes involved in producing Aβ [18]. This suggests that DPP4i may directly modulate the amyloidogenic pathway, further contributing to the reduction in Aβ burden.

In conclusion, the therapeutic benefits of DPP4i in AD appear to be multifaceted, involving modulation of insulin signaling, inflammation, oxidative stress, and the amyloidogenic pathway. These molecular changes likely contribute to the observed improvements in cognitive function and reduction in Aβ and tau pathology, highlighting the potential of DPP4i as disease-modifying agents for AD.

Limitations of existing research and future research directions

In this review study, we identified several limitations, which underscore the need for larger clinical studies and provide an opportunity for new avenues of research to be considered. These include long-term direct evaluation and implications of DPP4i in AD patients with and without T2DM, comparisons of medications within the class, and the need for larger clinical studies.

Predominantly Preclinical Insight

Preclinical research has substantially supported the idea that DPP4i have a neuroprotective effect, potentially slowing the progression of AD and improving cognitive function. These studies offer insights into this drug class's underlying mechanisms and potential benefits. However, variations in study design, animal models, specific DPP4i used, and outcome measures across these preclinical studies make it challenging to conclude and compare results definitively. It is important to note that as DPP4i have been evaluated in diverse AD models with varying genetic backgrounds, the findings could be valuable as the impact of DPP4i on various AD neuropathologies has been assessed.

Limited Clinical Evidence

There is a limited number and scope of clinical studies. Many are observational, lacking the rigor of randomized controlled trials. This limits the ability to establish a clear cause-and-effect relationship between DPP4i use and AD outcomes. Additionally, most clinical studies have relatively short follow-up periods. This makes it difficult to assess the long-term efficacy and safety of DPP4i in AD, especially when large-scale studies evaluating the long-term efficacy need to be assessed in AD patients with and without T2DM.

Lack of Standardized Outcome Measures

Studies lack standardized cognitive assessments and AD biomarkers, which can hinder comparisons and the ability to track disease progression consistently. With the new AD biomarkers approved, it will be important that future studies evaluating the impact of DPP4i include these biomarkers, which could help to assess the effect early in the state. 

Focus on Specific DPP4i

Most studies have focused on a few specific DPP4i (e.g., linagliptin, sitagliptin). However, more research is needed on other DPP4i to determine if there are class-wide effects or differences in efficacy between individual drugs.

## Conclusions

In conclusion, the multifaceted effects of DPP4i on Aβ and tau pathology, combined with their positive impact on cognitive function, neuroinflammation, oxidative stress, and neuronal survival, present a compelling case for their potential as disease-modifying agents for AD. Modifying insulin signaling, anti-inflammatory and antioxidant properties, GLP-1 upregulation, and modulation of the amyloidogenic pathway further strengthen their promise.

Although preclinical and clinical studies have shown encouraging results, further research is essential to fully unlock the therapeutic potential of DPP4i in AD. Future research must delve deeper into their long-term effects, safety profile, and optimal combination with other therapeutic approaches. This continued exploration holds the key to unlocking novel treatment options for AD, offering hope to millions affected by this devastating neurodegenerative disease.
